# Identification of salt gland-associated genes and characterization of a dehydrin from the salt secretor mangrove *Avicennia officinalis*

**DOI:** 10.1186/s12870-014-0291-6

**Published:** 2014-11-18

**Authors:** Pavithra A Jyothi-Prakash, Bijayalaxmi Mohanty, Edward Wijaya, Tit-Meng Lim, Qingsong Lin, Chiang-Shiong Loh, Prakash P Kumar

**Affiliations:** Department of Biological Sciences, National University of Singapore, 14 Science Drive 4, Singapore, Republic of Singapore; NUS Environmental Research Institute (NERI), National University of Singapore, #02-01, T-Lab Building, 5A Engineering Drive 1, Singapore, Republic of Singapore; Department of Chemical and Biomolecular Engineering, National University of Singapore, Singapore, Republic of Singapore; IFReC, Osaka University, 3-1 Yamada-oka, Suita, Osaka 565-0871 Japan; Temasek Life Sciences Laboratory, National University of Singapore, 1 Research Link, Singapore, Republic of Singapore

**Keywords:** *Avicennia officinalis*, Salinity, Dehydrin, Subtractive hybridization, Leaf salt glands, Drought stress

## Abstract

**Background:**

Salt stress is a major challenge for growth and development of plants. The mangrove tree *Avicennia officinalis* has evolved salt tolerance mechanisms such as salt secretion through specialized glands on its leaves. Although a number of structural studies on salt glands have been done, the molecular mechanism of salt secretion is not clearly understood. Also, studies to identify salt gland-specific genes in mangroves have been scarce.

**Results:**

By subtractive hybridization (SH) of cDNA from salt gland-rich cell layers (tester) with mesophyll tissues as the driver, several Expressed Sequence Tags (ESTs) were identified. The major classes of ESTs identified include those known to be involved in regulating metabolic processes (37%), stress response (17%), transcription (17%), signal transduction (17%) and transport functions (12%). A visual interactive map generated based on predicted functional gene interactions of the identified ESTs suggested altered activities of hydrolase, transmembrane transport and kinases. Quantitative Real-Time PCR (qRT-PCR) was carried out to validate the expression specificity of the ESTs identified by SH. A *Dehydrin* gene was chosen for further experimental analysis, because it is significantly highly expressed in salt gland cells, and dehydrins are known to be involved in stress remediation in other plants. Full-length *Avicennia officinalis Dehydrin1* (*AoDHN1*) cDNA was obtained by Rapid Amplification of cDNA Ends. Phylogenetic analysis and further characterization of this gene suggested that *AoDHN1* belongs to group II Late Embryogenesis Abundant proteins. qRT-PCR analysis of *Avicennia* showed up-regulation of *AoDHN1* in response to salt and drought treatments. Furthermore, some functional insights were obtained by growing *E. coli* cells expressing *AoDHN1*. Growth of *E. coli* cells expressing AoDHN1 was significantly higher than that of the control cells without AoDHN1 under salinity and drought stresses, suggesting that the mangrove dehydrin protein helps to mitigate the abiotic stresses.

**Conclusions:**

Thirty-four ESTs were identified to be enriched in salt gland-rich tissues of *A. officinalis* leaves. qRT-PCR analysis showed that 10 of these were specifically enriched in the salt gland-rich tissues. Our data suggest that one of the selected genes, namely, AoDHN1 plays an important role to mitigate salt and drought stress responses.

**Electronic supplementary material:**

The online version of this article (doi:10.1186/s12870-014-0291-6) contains supplementary material, which is available to authorized users.

## Background

*Avicennia officinalis* is an obligate halophyte that has evolved both morphologically and physiologically to thrive in saline conditions [1]. Multicellular salt glands are found on *A. officinalis* leaves that help to secrete excess salt, which is one of the key adaptations leading to salt tolerance of these plants [1-6]. Some studies have shown that salt secretion is an energy dependant process [4], while others have indicated that it can occur through exocytosis [7,8]. Although a large number of studies have been conducted on the structure of salt glands [3,6,9-12], only a few were regarding their function [13]. Therefore, studies such as identification of genes that are specifically expressed in salt glands will contribute significantly towards resolving mangrove salt gland function.

Over the last decade many techniques have been developed to identify genes that are specifically or preferentially expressed in the tissue of interest [14-18]. Subtractive hybridization (SH) is one such tool [19], which has been widely used in various organisms including plants [18,20,21]. Despite several transcriptomic studies carried out to identify genes responsible for salt tolerance in other mangroves such as *Bruguiera* and *Aegiceras* [22-24], the molecular mechanisms regulating salt secretion have not been established so far. The mangrove salt glands occur primarily on the leaf epidermis. Hence, the use of isolated epidermal peels that are salt gland-rich will increase the probability of identifying genes expressed preferentially in the glands [25]. Therefore, SH technique could be exploited to identify genes that are expressed in salt gland-rich tissues of the mangrove *A. officinalis*.

In addition to salt secretion, production of osmolytes [26-28] or specialized proteins such as Late Embryogenesis Abundant (LEA) proteins has been shown to protect macromolecules in the cells under stress [29,30]. A special class of LEA proteins (group II) known as dehydrins has been shown primarily to play important roles in alleviating salt and other abiotic stresses through their protective action by binding to macromolecules [29,31-35]. Dehydrins are intrinsically unstructured proteins that contain three conserved motifs: Y, S and K and are divided into five subgroups [36]. Each subgroup has been identified to play a role in response to a specific abiotic stress condition [31]. Mangroves such as *Avicennia marina* have been shown to contain dehydrins [37]. Nevertheless, the role of dehydrins in mangrove salt glands has not been adequately understood yet. Although, dehydrins have been identified from mangroves such as *Avicennia marina*, their occurrence in salt glands and role in salt secretion have not been well explored.

In this study, we have identified differentially expressed genes in salt gland-rich leaf tissues of *Avicennia officinalis* using SH technique. We have generated a predicted functional gene interaction map of *A. officinalis* salt glands using the identified ESTs. Additionally, quantitative RT-PCR validation of several ESTs that are preferentially expressed in the salt glands compared to mesophyll tissue has also been carried out. Here we report characterization of a *Dehydrin* gene (*AoDHN1*) identified from the SH analysis. Its expression pattern and response to salinity and drought stress treatments were studied in *A. officinalis*. We present data suggesting the abiotic stress-mitigating function of AoDHN1 by growing *E. coli* cells expressing *AoDHN1* under salinity and drought stresses. Taken together, our data suggest that *AoDHN1* plays an important role in salt and drought stress remediation in *A. officinalis*.

## Results

### Classification of differentially expressed ESTs and expression analysis of selected ESTs

**Figure 1:**
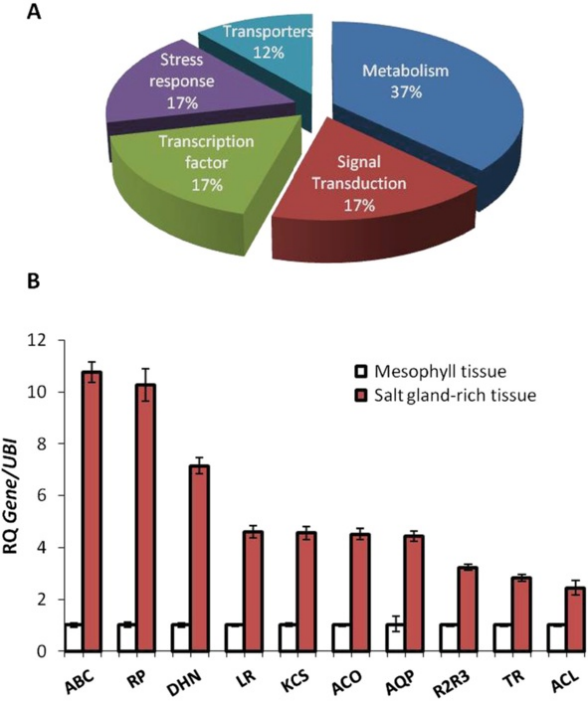
**Classification of differentially expressed ESTs and expression analysis of selected ESTs. (A)** Distribution of ESTs obtained from subtractive hybridization of salt gland rich-tissue and mesophyll tissue from *A. officinalis* leaves. **(B)** Expression profile of selected EST’s enriched in salt glands by qRT-PCR analysis of transcripts from mesophyll tissue vs. salt gland rich-tissue. *White-brown-complex ABC transporter family (ABC), Ribosomal protein S6 (RP), Dehydrin (DHN), Leucine-rich repeat protein kinase (LR), 3-ketoacyl-CoA synthase (KCS), 1-aminocyclopropane-1-carboxylate oxidase (ACO), Aquaporin (AQP), Transcription factor R2R3 (R2R3), Thioredoxin H (TR), ATP Citrate Lyase (ACL)* RQ – Relative quantification (mean ± SE, n = 3).

From the subtracted cDNA library, we identified 900 ESTs. Most of the ESTs identified could not be annotated based on function, hence they were classified as unknown and were omitted from further analysis. Among the annotated ESTs, 62 showed high *e*-values and upon removing the duplicates, 34 unique ESTs were obtained. These were then grouped under several categories (Figure 1) based on predicted functions (Table 1).

**Table 1:** ***Avicennia officinalis*** **ESTs identified from salt gland-rich tissue after subtractive hybridization**

***Avicennia*** **clone ID and classification**	**Putative function**	**O**_**f**_	**Reference organism and accession no.**	***Avicennia officinalis*** **EST GenBank accession no.**	***e*** **-value**
704864 Signal transduction	Mitochondrial Rho GTPase	1	*Glycine max* GLYMA10G29580.1	JZ721695	9.00E-71
719405 Signal transduction	Leucine-rich repeat protein kinase	1	*Arabidopsis thaliana* AT5G49760.1	JZ721696	4.00E-80
719420 Signal transduction	Serine/threonine-protein kinase	1	*Glycine max* GLYMA07G30791.1	JZ721697	3.00E-57
720115 Signal transduction	Casein kinase II, alpha chain, putative	1	*Oryza sativa* LOC_Os07g02350.1	JZ721679	4.00E-100
720108 Signal Transduction	Xylem cysteine peptidase 2	1	*Arabidopsis thaliana* AT1G20850.1	JZ721680	1.00E-26
708681 Signal transduction	Serine/arginine-rich protein splicing factor 34b	1	*Arabidopsis thaliana* AT4G02430.1	JZ721698	8.00E-50
721424 Metabolism/Amino acid	Trypsin family protein	1	*Arabidopsis thaliana* AT5G45030.1	JZ721699	1.00E-95
694067 Metabolism/Amino acid	Glutamate synthase	2	*Oryza sativa* LOC_Os01g48960.1	JZ721681	4.00E-107
708680 Metabolism/Amino acid	Arginine decarboxylase	1	*Populus trichocarpa* POPTR_0004s17020.1	JZ721700	3.00E-81
708683 Metabolism/Lipid	ATP-citrate lyase	1	*Arabidopsis thaliana* AT1G60810.1	JZ721701	0
694059 Metabolism/Lipid	Phospholipase D	1	*Glycine max* GLYMA07G03490.3	JZ721702	3.00E-160
719630 Metabolism/Lipid	3-ketoacyl-CoA synthase	4	*Glycine max* GLYMA04G20620.1	JZ721682	4.00E-48
719448 Metabolism/Protein	Protein translation factor SUI1 homolog	1	*Oryza sativa* LOC_Os07g34589.2	JZ721683	6.00E-83
714704 Metabolism/Protein	Syringolide-induced protein 19-1-5	1	*Glycine max* GLYMA17G07240.1	JZ721703	4.00E-15
720030 Metabolism/Protein	Ribosomal protein S6	1	*Arabidopsis thaliana* AT4G31700.1	JZ721710	1.00E-154
719373 Metabolism/Sugar	Trehalose 6-phosphatase synthase S6	1	*Arabidopsis thaliana* AT1G68020.1	JZ721704	1.00E-50
720067 Metabolism/Vitamin	1-aminocyclopropane-1-carboxylate oxidase	1	*Populus trichocarpa* POPTR_0002s21750.1	JZ721705	1.00E-151
719392 Metabolism/Energy	Cytochrome-c oxidase	1	*Arabidopsis thaliana* AT1G80230.1	JZ721688	1.00E-46
703868 Metabolism/Energy	Thioredoxin H	7	*Avicennia marina* BM497420.1	JZ721687	8.00E-143
703936 Stress response	Ubiquitin-conjugating enzyme 2	1	*Arabidopsis thaliana* AT2G02760.1	JZ721684	4.00E-52
719444 Stress response	26S protease regulatory subunit 4 homolog	1	*Oryza sativa* LOC_Os07g49150.1	JZ721685	2.00E-119
719392 Stress response	Dehydrin	9	*Avicennia marina* BM172730.1	JZ721686	9.00E-42
703860 Stress response	Peroxidase	1	*Avicennia marina* BM173160.1	JZ721712	1.00E-175
724765 Stress response	Disease resistance	1	*Avicennia marina* BM497281.1	JZ721711	1.00E-65
704843 Transcription factor	NAC domain containing protein 32	4	*Arabidopsis thaliana* AT1G77450.1	JZ721689	1.00E-24
709313 Transcription factor	Transcription factor R2R3 factor gene family	3	*Arabidopsis thaliana* AT3G12720.1	JZ721690	2.00E-13
719394 Transcription factor	Auxin signaling F-box 2	1	*Arabidopsis thaliana* AT3G26810.1	JZ721706	7.00E-45
720062 Transcription factor	Salt-inducible zinc finger 2	1	*Arabidopsis thaliana* AT2G40140.1	JZ721691	9.00E-41
713984 Transcription factor	Transcription factor HBP-1b	1	*Oryza sativa* LOC_Os01g59350.1	JZ721707	2.00E-22
714010 Transcription factor	AP2 domain-containing transcription factor	1	*Populus trichocarpa* POPTR_0016s08530.1	JZ721708	3.00E-73
694065 Transporter/ABC	White-brown-complex ABC transporter family	4	*Arabidopsis thaliana* AT1G51460.1	JZ721692	5.00E-23
720073 Transporter/Ion	Vacuolar ATP synthase subunit D	1	*Arabidopsis thaliana* AT3G58730.1	JZ721693	2.00E-108
709299 Transporter/Ion	Plasma membrane H + ATPase	1	*Avicennia marina* BM172881.1	JZ721694	6.00E-179
719615 Transporter/Water	Aquaporin	3	*Oryza sativa* LOC_Os04g47220.1	JZ721709	7.00E-119

Functional annotation was done after blasting the sequences with various plant gene databases. Clone ID with classification (column 1) and the putative function (column 2) based on comparison with reference organisms are shown. Occurrence frequency (O_f_), which is the number of times a specific EST was identified in the SH is given in column 3. The reference organism to which the EST was compared with and its accession number are given in columns 4 and *Avicennia officinalis* EST GenBank accession numbers are given in column 5. The *e*-values of sequence comparison of the *A. officinalis* ESTs with the reference sequences are given in column 6.

The major classes of genes obtained from SH corresponded to metabolism (37%), stress response (17%), signal transduction (17%), transcription factor (17%) and transporters (12%) (Figure 1A). Genes involved in lipid, amino acid and carbohydrate metabolic pathways were identified. Among the stress responsive classes of genes, those involved in protein recycling, namely, ubiquitin conjugating enzyme and 26S proteasome regulatory subunit were abundant (Table 1). The transporter genes identified included *Aquaporins*, *ATP-Binding Cassette* (ABC) transporter family, *Vacuolar ATP synthase subunit* and *Plasma membrane H*^*+*^*-ATPase*. Several kinase genes, including *Casein Kinase*, *Serine/Threonine Kinases* along with *GTPase* were identified in the signal transduction class. *NAC Domain-containing Protein 32*, transcription factor *R2R3*, *F-box 2*, *Salt-inducible Zinc Finger* are some of the transcription factor genes that were identified in transcription factor class.

Tissue-specificity of expression of the 34 selected ESTs was verified by qRT-PCR (Figure 1B and Additional file 1). *ABC transporter* and *Ribosomal protein S6* showed more than 10-fold abundance in salt gland-rich tissue compared to mesophyll tissue. A *Dehydrin* gene identified from SH (*AoDHN1*) showed more than 6-fold increase (Figure 1B). Of these, *Dehydrin* is a gene with a possible function relevant for abiotic stress tolerance. Hence, it was a preferred gene for further studies. The remaining ESTs, namely, *Leucine-Rich Repeat Receptor*, *3-Ketoacyl-CoA Synthase*, *1-Amino-Cyclopropane-1-Carboxylate Oxidase* (*ACC Oxidase*) and *Aquaporin* showed ~5-fold higher expression in the salt gland-rich tissue. *R2R3* transcription factor, *Thioredoxin H* and *ATP Citrate Lyase* showed about 3- to 4-fold higher expression in the salt gland-rich tissue. Expression analyses of other ESTs which showed no significant differential expression are provided in Additional file 1.

### Functional gene-network analysis of the ESTs identified from Subtractive Hybridization

**Figure 2:**
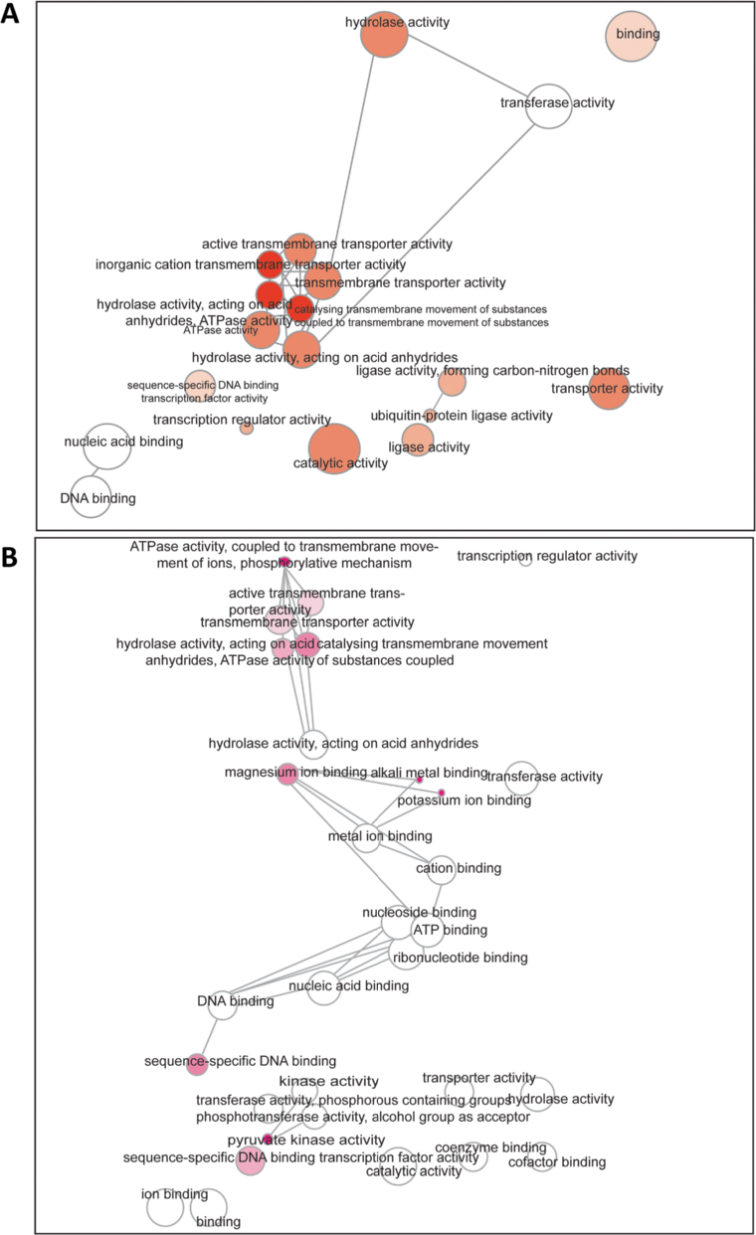
**Functional gene-network analysis of the ESTs identified from subtractive hybridization.** Interactive graph was generated using web-tool REVIGO (http://revigo.irb.hr/) as on 9^th^ December 2013. The bubble colour indicates the p-value as generated by Singular Enrichment Analysis of the Gene Ontology (GO) terms obtained from the web-tool agriGO (http://bioinfo.cau.edu.cn/agriGO/analysis.php). The gene IDs that resulted by blasting the ESTs against **(A)** *Arabidopsis* and **(B)** Poplar cDNA libraries were used to generate the GO terms. Bubble size indicates the frequency of the GO term. Highly similar GO terms are linked by edges in the graph, where the line width indicates the degree of similarity.

An interactive REVIGO (REduce VIsualize Gene Ontology) graph that indicates the functional network of the identified ESTs was generated [38]. The overview of this graph shows a functional gene network in salt gland-rich tissue generated against *Arabidopsis* cDNA library. At the center of the network, a tight cluster of interaction between hydrolase, ATPase and transmembrane transport activity is depicted (Figure 2A). Transmembrane transport activity seemed to be coupled with ATPase and hydrolase activities. Ligase activity is seen further down the gene network, especially the activity of ubiquitin-protein ligase. Although extensive transmembrane transport and hydrolase activities seem to be occurring in a narrow-range, transferase activity, nucleic acid binding and sequence-specific DNA binding transcription factor activities were also observed.

Similarly, the interactive graph developed by comparison with poplar cDNA database also highlights ATPase, hydrolase and transmembrane transport activities (Figure 2B). A tight cluster of these three activities was observed but with lower intensity. In parallel, tiny clusters of nucleic acid binding and metal ion binding activities were observed. Magnesium ions, alkali metal ion, potassium ion, cation binding activities were major ion binding clusters. However, sequence-specific DNA binding, nucleotide-, nucleoside- and ATP-binding were included in the nucleic acid binding clusters. An additional small binding cluster of phosphotransferase that are involved in kinase activity was also observed.

### cDNA and genomic DNA sequences of *AoDHN1*

**Figure 3:**
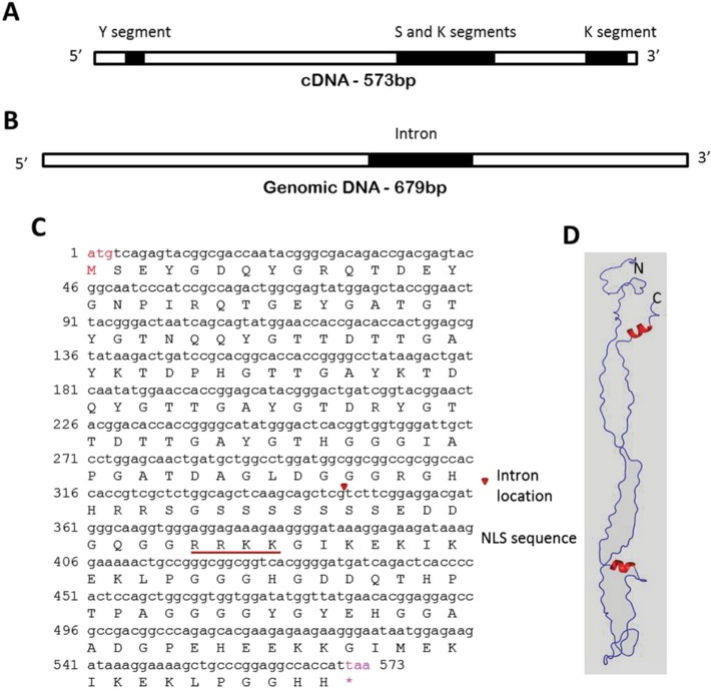
**cDNA and genomic DNA sequences of** ***AoDHN1.*** **(A)** cDNA of 573 bp corresponding to Open Reading Frame (ORF) of *AoDHN1* obtained from Rapid Amplification of cDNA Ends (RACE) PCR. Y, S and two of K segments are depicted on the ORF. **(B)** Genomic fragment of *AoDHN1* with intron of 107 bp. **(C)** Nucleotide sequence of *AoDHN1* and its corresponding translated protein sequence. Arrowhead indicates intron location and underline indicates Nuclear Localization Signal (NLS) sequence. **(D)** Predicted three dimensional structure of AoDHN1 obtained using iTASSER server (http://zhanglab.ccmb.med.umich.edu/I-TASSER/) showing two alpha helices (in red), but the rest of the molecule is unstructured.

Subtractive hybridization of *A. officinalis* led to the identification of an EST (*AoDHN1*) that showed homology to *Avicennia marina Dehydrin1* (*AmDHN1*). The coding sequence of *AoDHN1* is 573 bp and the corresponding genomic sequence is 679 bp, because of the presence of an intron (106 bp) (Figure 3A). The cDNA sequence stretch coding for a single uninterrupted polypeptide of 190 amino acids was identified by *in silico* translation of the sequence corresponding to AoDHN1, with a deduced molecular mass of 19.82 kDa. A nuclear localization sequence (NLS) RRKK has been identified towards the C-terminus of AoDHN1 suggesting that it could be a nuclear-localized protien (Figure 3C). Also, the location of the intron has been identified in the genomic DNA sequence (Figure 3C). The predicted two-dimensional structure of the dehydrin proteins using PSIPRED revealed a major unstructured region and two possible α-helices (see Additional file 2). Additionally, the three-dimensional structure of AoDHN1 generated using iTASSER confirmed the presence of the unstructured region along with two α-helices (Figure 3D).

### Classification of AoDHN1 as a Group II LEA protein

**Figure 4:**
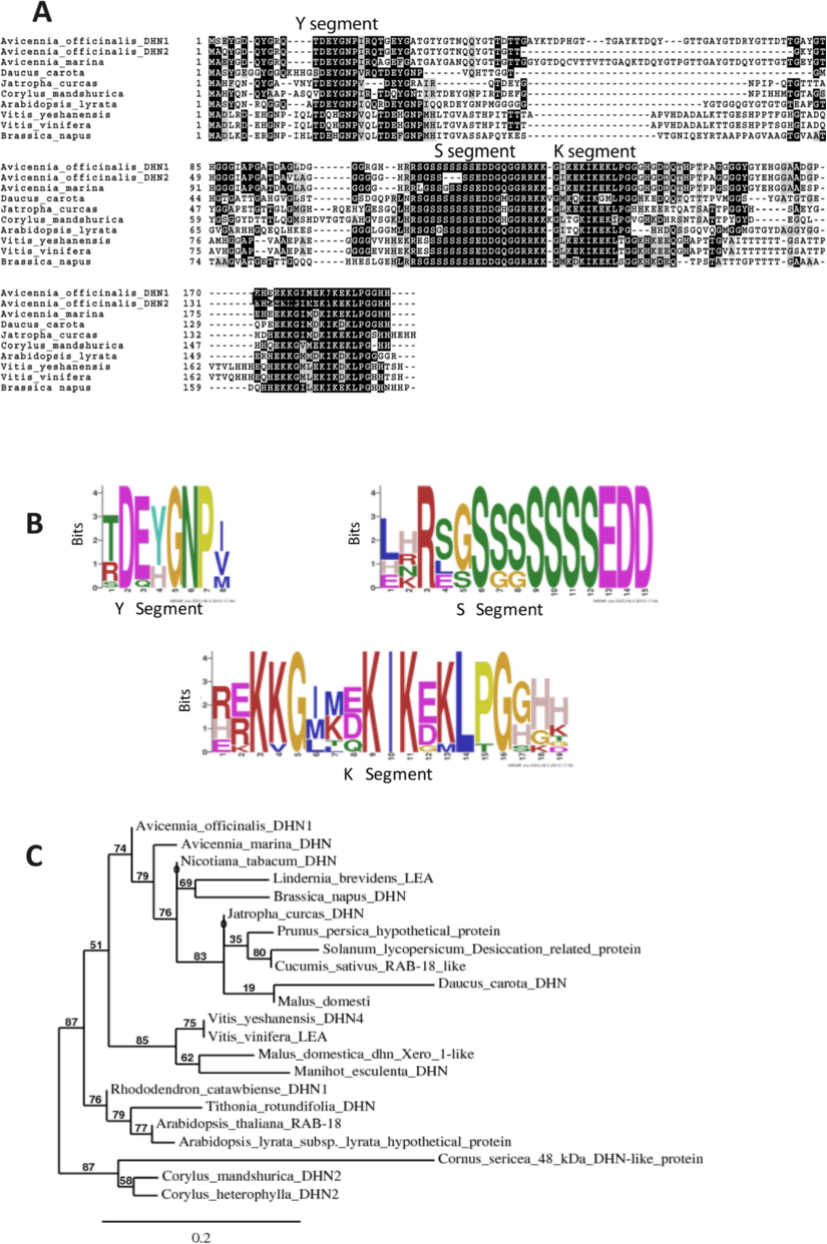
**Classification of AoDHN1 into Group II LEA protein based on sequence alignment and phylogenetic analysis. (A)** Alignment of AoDHN1 and AoDHN2 protein sequences with dehydrins from other plant species. The shaded region shows the conserved motif YSK2 (http://www.ch.embnet.org/software/BOX_form.html). **(B)** Conserved sequence motifs identified from AoDHN1 using MEME web-tool (http://meme.nbcr.net/meme/). Amino acid pattern that occurs repeatedly in YSK2 family dehydrins are represented in position-dependent manner. **(C)** The phylogenetic relationship of AoDHN1 with group II LEA proteins of different species is represented in rooted dendrogram. It was constructed using Phylogeny.fr web-tool (http://phylogeny.lirmm.fr/phylo_cgi/simple_phylogeny.cgi) by the approximate likelihood method based on a complete protein sequence alignment of different dehydrins and the approximate likelihood-ratio test. The branch support values are shown at the nodes as percentage values and scale bar indicates the branch lengths. The gi numbers for the sequences are: |*gb*|KM652423| *AoDHN1 [Avicennia officinalis]; gi|157497151|gb|ABV58322.1| dehydrin [Avicennia marina]; gi|349844874|gb|AEQ19906.1| dehydrin 4 [Vitis yeshanensis]; gi|225428392|ref|XP_002283605.1| PREDICTED: late embryogenesis abundant protein-like [Vitis vinifera]; gi|353685443|gb|AER13140.1| DHN2 [Corylus mandshurica]; gi|307776652|gb|ADN93460.1| dehydrin 2 [Corylus heterophylla]; gi|314998614|gb|ADT65201.1| dehydrin [Jatropha curcas]; gi|449457626|ref|XP_004146549.1| PREDICTED: dehydrin Rab18-like [Cucumis sativus]; gi|442022395|gb|AGC51773.1| dehydrin protein [Manihot esculenta]; gi|34539778|gb|AAQ74768.1| dehydrin [Brassica napus]; gi|657980608|ref|XP_008382297.1| PREDICTED: late embryogenesis abundant protein [Malus domestica]; gi|57506540|dbj|BAD86644.1| dehydrin protein [Daucus carota]; gi|15239373|ref|NP_201441.1| dehydrin Rab18 [Arabidopsis thaliana]; gi|472278804|gb|AGI37442.1| dehydrin 1 [Rhododendron catawbiense]; gi|18076154|emb|CAC80717.1| putative dehydrin [Tithonia rotundifolia]; gi|595807384|ref|XP_007202596.1| hypothetical protein PRUPE_ppa011637mg [Prunus persica]; gi|297794373|ref|XP_002865071.1| hypothetical protein ARALYDRAFT_496967 [Arabidopsis lyrata subsp. lyrata]; gi|19032422|gb|AAL83427.1|AF345989_1 48 kDa dehydrin-like protein [Cornus sericea]; gi|657948498|ref|XP_008338082.1| PREDICTED: dehydrin Xero 1-like [Malus domestica]; gi|129562715|gb|ABO31098.1| late embryogenesis abundant protein [Lindernia brevidens]; gi|46020012|dbj|BAD13498.1| dehydrin [Nicotiana tabacum]; gi|460373256|ref|XP_004232437.1| PREDICTED: desiccation-related protein clone PCC6-19-like isoform 2 [Solanum lycopersicum].*

Sequence alignment with group II LEA proteins of other plant species showed that AoDHN1 belongs to YSK2 sub-class of dehydrins (Figure 4A). The amino acids from 12 to 18 (TDEYGNP) correspond to the Y segment, while amino acids from 107 to 124 correspond to the S segment, and there are two K segments stretching from amino acids 129 to 141 and 173 to 187. Because this dehydrin possesses one Y, one S and two K segments, it is named as the YSK2 sub-group (Figure 4A and 4B). Only the domain-specific regions (YSK2) show consensus between AoDHN1 and other dehydrins (group II LEA proteins). AoDHN1 shows a high similarity index of 84% with AmDHN1 and both the dehydrins were found to be closely related based on phylogenetic analysis (Figure 4C).

### *AoDHN1* copy number in the genome

**Figure 5:**
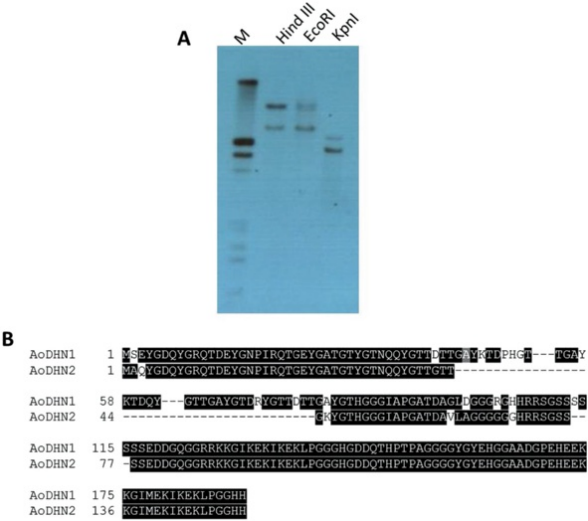
***AoDHN1*** **copy number in the genome. (A)** Genomic Southern blot showing two copies of *AoDHN1* in *Avicennia officinalis.* **(B)** Alignment of AoDHN1 and AoDHN2 (dehydrin obtained from transcriptome sequencing) using ClustalW2 multiple alignment (http://www.ebi.ac.uk/Tools/msa/clustalw2/) and represented using the web-tool BoxShade Server (http://www.ch.embnet.org/software/BOX_form.html).

A full length gene probe showed two copies of *Dehydrin* in a genomic Southern blot analysis (Figure 5A). On examining the sequence similarity with *Dehydrin* sequences obtained in our lab from *A. officinalis* transcriptome analysis (unpublished data), it was found that another *Dehydrin* (*AoDHN2*) sequence shared high similarity with *AoDHN1* (Figure 5B). This confirmed the identification of two *Dehydrins* in the genome of *A. officinalis*.

### Characterization of *AoDHN1*

**Figure 6:**
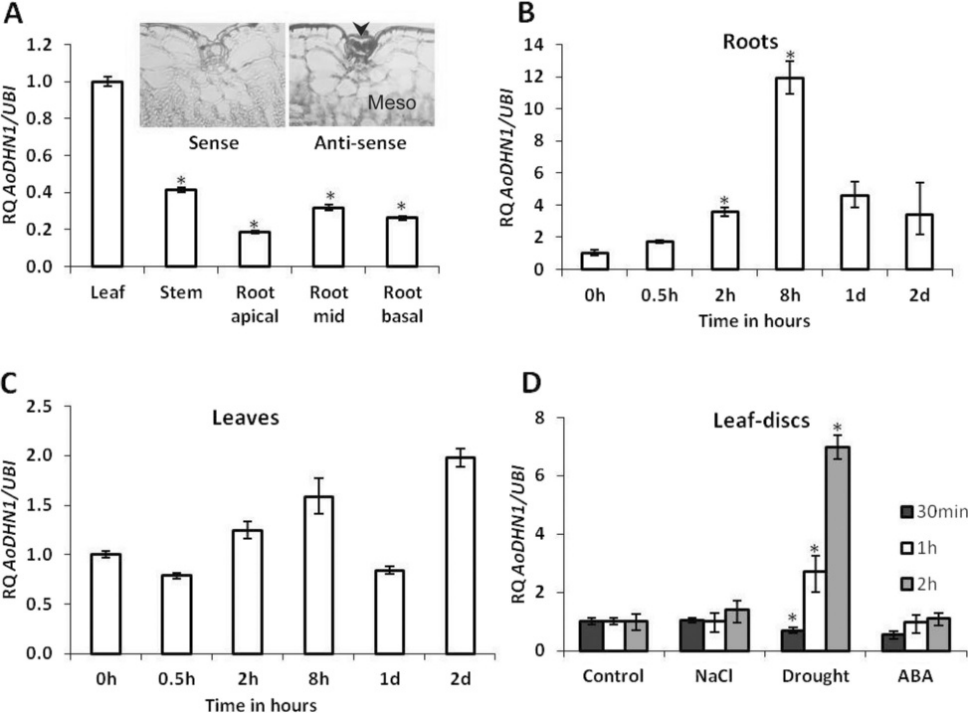
**Expression profile of** ***AoDHN1.*** **(A)** Tissue-specific expression of *AoDHN1* transcripts from two*-*month-old greenhouse-grown plants. (Inset to A) *In situ* hybridization of leaf tissue, showing high abundance of *AoDHN1*expression in the salt glands (n = 3). Arrowhead indicates the salt gland, and mesophyll cells are labeled as Meso. **(B)** Expression kinetics of *AoDHN1* upon salt stress in roots. **(C)** Expression kinetics of *AoDHN1* upon salt stress in leaves. **(D)** Expression analysis of *AoDHN1* in the *A. officinalis* leaf-discs upon treatment with salt (NaCl), drought and ABA. Asterisks indicate a significant difference in expression levels as indicated by Student’s *t*-test (*p* < 0.05). RQ-Relative quantification data representing mean ± SE (n = 3).

Tissues collected from two-month-old seedlings that were not exposed to salt were used for tissue-specific expression analysis. The highest expression of *AoDHN1* was observed in the leaves compared to roots (root apical, root mid and root basal) and stems (Figure 6A). *In situ* hybridization studies from leaves of two-month-old *A. officinalis* seedlings confirmed abundant expression of *AoDHN1* in salt glands (Figure inset of 6A). Expression kinetics of *AoDHN1* was tested in both roots and leaves of *A. officinalis* seedlings upon salt treatment (Figure 6B and 6C). A 10-fold increase in expression levels of *AoDHN1* in the roots was seen after 8 h while a 2-fold increase was seen in the leaves after 48 h of salt treatment.

Leaf discs from two-month-old seedlings (previously not exposed to salt) were chosen to study the regulation of *AoDHN1* by abiotic stresses (Figure 6D). Drought treatment for 1 h and 2 h showed a 2- and 6-fold increase respectively, in the expression of *AoDHN1*. However, abscisic acid (ABA) and salt treatments did not affect the expression of *AoDHN1* up to 2 h.

**Figure 7:**
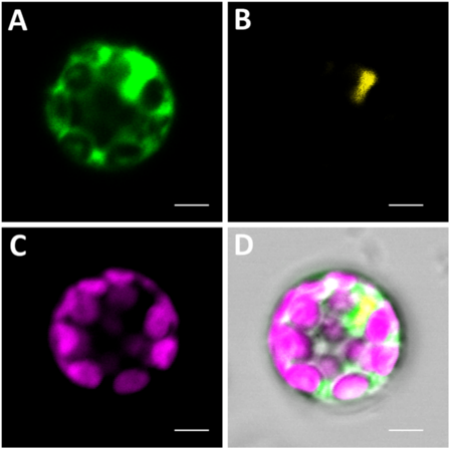
**Sub-cellular localization of GFP fused AoDHN1 in** ***Arabidopsis*** **mesophyll protoplasts. (A)** Localization of AoDHN1-GFP in the cytoplasm and nucleus **(B)** Position of the nucleus is indicated by yellow fluorescence from YFP fused with nuclear localization signal of SV40 (NLS_SV40_-YFP) **(C)** Auto fluorescence of chloroplasts (artificially coloured purple) **(D)** Merged image of **(A) (B) (C)** and **(D)** overlaid on transmitted light image of the protoplast. Scale bar = 5 μm.

Transient expression of *35S::AoDHN1-GFP* construct transfected into *Arabidopsis* mesophyll protoplasts showed the localization of AoDHN1-GFP fusion protein in the cytosol as well as the nucleus (Figure 7). Yellow fluorescence from YFP fused with the nuclear localization signal of SV40 was used to detect the nucleus.

### Functional assay of *AoDHN1* in *E. coli* cells

**Figure 8:**
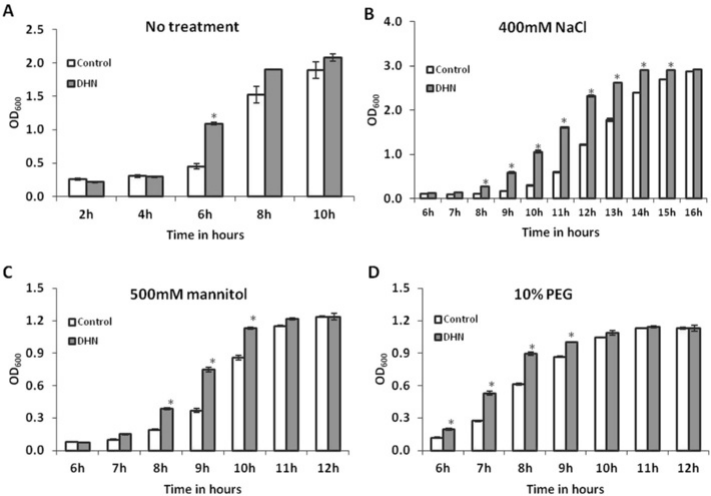
**Comparison of growth of** ***E. coli*** **cells expressing AoDHN1 under salt and drought stress conditions.** Salt stress was simulated by 400 mM NaCl, and drought conditions were provided by 500 mM mannitol and 10% polyethylene glycol (PEG). *E. coli* BL21 cells expressing AoDHN1 showed significant differences in cell densities compared to control cells which do not express AoDHN1 over specified periods of time. **(A)** without any treatment **(B)** with 400 mM NaCl treatment **(C)** with 500 mM mannitol treatment and **(D)** with 10% PEG 4000 treatment. Data are mean ± SE (n = 3). Asterisks indicate a significant difference in cell densities as indicated by Student’s *t*-test (*p* < 0.05). OD – Optical Density.

Salinity (NaCl) and drought (mannitol and polyethylene glycol 4000 - PEG) stress response of AoDHN1 in *E. coli* bacteria was tested. The *E. coli* (BL21 cells) transfected with *pGEX-6p-1-AoDHN1* and empty vector separately, were subjected to 400 mM NaCl, 500 mM mannitol and 10% PEG treatment. A control study was done without any treatment to check the difference in growth between *E. coli* cells transfected with *pGEX-6p-1-AoDHN1* and empty vector. OD_600_ of the bacterial culture was taken at 2 h time intervals after induction of AoDHN1 expression by IPTG-treatment. The *E. coli* cells expressing AoDHN1 showed better growth (as represented by higher cell density) compared to the control after 6 h (Figure 8A) even in the absence of any treatment. With NaCl treatment, the cell densities started to show significant differences from 8 h onwards (Figure 8B). Upon mannitol treatment, *E. coli* cells expressing AoDHN1 showed significantly higher growth between 8 h and 10 h (Figure 8C). On the other hand, with PEG treatment, the difference in growth was apparent from 6 h and lasted up to 9 h (Figure 8D). Therefore, the protective function of AoDHN1 protein was demonstrated by the growth advantage conferred under salinity and drought stresses for *E. coli* cells expressing *AoDHN1*.

## Discussion

Salt secretion is a dynamic and energy dependent process as shown in *Avicennia* species [9,39]. Identification of genes that are expressed in salt glands will help in understanding the secretion process. While many genes related to salt tolerance have been identified using SH and transcriptome analysis from the leaves of other mangrove species [22-24,40-43], there have been no attempts to specifically identify the genes that are expressed in salt glands.

A meaningful way of analysing SH data obtained from our experiment was to create a network using REVIGO, of the ESTs of *A. officinalis* against *Arabidopsis* and poplar cDNA libraries which would give an overview of functional gene interaction in salt gland-rich tissue [38]. This collection of ESTs from salt gland-rich tissue depicts potential interaction between gene products either with each other or with other molecules in the cell, thereby suggesting the global functional network. The interactive Gene Ontology (GO) map of the ESTs with both *Arabidopsis* and poplar cDNA libraries suggests that activities of hydrolase, transmembrane transport, nucleotide binding and kinase functions are common in the selected tissue (Figure 2A and 2B). Transmembrane transport includes channels, pumps and transporters, which are important in maintaining ion homeostasis and contribute to salt tolerance. Ion transporters like H^+^-ATPases, V-ATPases and SOS1(Salt Overly Sensitive1) are known to bring about ionic balance in the cell [26], while transporters like aquaporins stabilize water movement and contribute to osmotic regulation [9]. Kinases identified from our study belong to Receptor-Like Kinases (RLKs) which regulate several plant processes such as growth, development and homeostatic mechanisms intrinsic to abiotic stress response [44]. This visual outline aids in understanding the possible functional relations of the identified ESTs from salt gland-rich tissue of *A. officinalis*.

### Identification of genes that are highly expressed in salt gland-rich tissue

Aquaporins and ABC transporters were the major transporters identified in our study. ABC transporters are known to transport fatty acids that are required for proper cuticle development in leaves [45]. The cuticle plays an important role in maintaining the structural integrity of salt glands. Under saline conditions, it becomes important for the salt glands to form a thick cuticular layer to prevent water loss and also diffusion of ions into neighbouring cells [6]. The observed high level of expression of ABC transporters in the salt gland-rich tissue could explain this in *Avicennia* salt gland cells. Aquaporins are known to regulate water movement across the membranes. During drought and salt exposure, aquaporins are known to maintain water balance in the cells [46] and have been shown to play a crucial role in salt secretion of *A. officinalis* [9]. Although aquaporins have been identified from the leaves of other salt secretors [41], its precise function in regulating water movement during secretion is not clear. Another major class of ESTs identified was related to metabolic processes. Physiological response of the plant is known to be altered due to metabolic changes under salt stress. Abiotic stresses usually cause energy deprivation, therefore plants tend to regulate metabolic genes by suppressing genes encoding biosynthetic enzymes of amino acids to conserve energy, and induce genes coding catabolic enzymes of amino acids to provide energy [47]. Cysteine peptidase identified from our SH study is known to play a role in plant stress response by participating catabolic process [48]. Ethylene plays an important role during salt stress [49] and ethylene biosynthesis is regulated by the levels of ACC oxidase activity [50], which is reported to have a positive effect on salinity tolerance [51]. The roles played by these genes in salt secretion and tolerance are still not clear, but the complexity of the underlying metabolic processes is highlighted. Our observations provide further evidence to the view that a combined action of different metabolic pathways and active expression of various transporters would have to be occurring in order to maintain cellular homeostasis under stress.

Among the class of stress-related proteins, a dehydrin was identified. Dehydrins are hydrophilic and highly flexible proteins (Intrinsically Unstructured Proteins, IUPs) that protect cytosolic and membrane proteins by wrapping around them [52] (‘molecular-huggers’). They seem to function similar to chaperones by stabilizing the protein folding, but dehydrins are much smaller in size and are less complex compared to chaperones. Because they belong to IUPs, it has been quite challenging to determine their precise function through structural studies. However, due to their significant association with protecting proteins against physiological drought, dehydrin was chosen for further examination in our study.

Dehydrins have been identified in a wide variety of organisms such as bacteria, chironomid, brine shrimp, nematodes, rotifers and cyanobacteria in response to desiccation [53], and are well-studied in plants for their key role in response to abiotic stress. Like group I LEA proteins, several studies of specific group II LEA proteins have confirmed that they accumulate during seed desiccation and in response to water deficit induced by drought, low temperature, or salinity [54,55].

A characteristic feature of group II LEA proteins is the presence of conserved domains such as Y, S and K. The K-segment consists of Lys-rich 15-residue motif, EKKGIMDKIKEKLPG [32], the Y-segment consensus sequence is [V/T]D[E/Q]YGNP [31,56] and serine rich S-segment contains LHRSGS4–10(E/D)3, which in some proteins can be phosphorylated [57]. The ORF encoded by *AoDHN1* has a single ‘Y’ segment, characterized by the presence of amino acids ‘DEYGNP’ followed by a serine-rich tract and two lysine-rich ‘K’ segments (Figure 4), and hence it belongs to the YSK2 class of dehydrins [58]. This is the most abundant class of dehydrins and is known to be induced by ABA and drought, but not cold temperatures [58]. *AmDHN1* is the only other mangrove dehydrin studied that belongs to YSK2 class of proteins [37] while a K-type dehydrin has been identified in *Rhizophora mucronata* [59]. However, our study is the first one to report that AoDHN1 is preferentially expressed in the salt gland cells.

### Expression and regulation of *AoDHN1*

Dehydrins are present in most of the vegetative tissues under optimal growth conditions [33]. Dehydrins from *Arabidopsis*, *Craterostigma* and *Citrus* have been shown to prevent inactivation of enzymes induced by partial dehydration in vitro [36,60]. Although some of the previous reports showed that dehydrins are expressed in all parts of the plants, the present study provides evidence for the preferential expression of *AoDHN1* in *A. officinalis* salt glands (Figure 1B, 6A and inset of 6A). Studies from several plant species indicated that different types of group II LEA proteins are present in various tissue types [55,61], which explains the preferential expression of *AoDHN1* observed in our study. Moreover, the unstructured nature of dehydrins was suggested to allow them to maintain enough water molecules in the cellular microenvironment and thus stabilize the macromolecules during water scarcity [36]. Since salt glands are the main site of secretion, there will be high concentration of ions in the cells, and hence it is conceivable that the macromolecules within those cells might require the protective action provided by dehydrins. It is therefore not a coincidence to find high level of *AoDHN1* expression in the salt glands as observed in our study. Further studies are needed to understand the exact mechanism by which this protective function is conferred within the salt gland cells.

Dehydrins are known to accumulate in every tissue upon water deficit imposed by abiotic stresses, such as drought and salinity [33]. A group II LEA gene from rice (*Oryza sativa*) was found to be specifically ABA-responsive and not directly responsive to salt stress [62]. While in sunflower, elevated *dehydrin* transcript levels appeared independent of ABA content in late embryogenesis [61] and *paf93* which belongs to dehydrin family from barely did not respond to exogenous ABA treatments [60]. Hence, we decided to examine if *AoDHN1* has specificity in its response to salinity, drought and ABA in *A. officinalis* leaf discs subjected to these stresses. The observed activation of *AoDHN1* in response to drought (Figure 6D) and salt (Figure 6B and 6C), but not ABA treatment (Figure 6D) suggests AoDHN1 responds to the abiotic stresses independently of ABA. These observations are in agreement with some of the earlier reports [63,64].

A majority of group II LEA proteins accumulate in the cytoplasm while some of them have been shown to localize in the nucleus as well [65]. Our data showed that GFP-fused AoDHN1 was transiently expressed in *Arabidopsis* mesophyll protoplasts, the protein was present in both the cytosol and nucleus, which is similar to the observation with AmDHN1 from *A. marina* [37] and Rab17/DHN1 from maize [57]. This suggests that AoDHN1 may function as non-specific protectant by binding to proteins in the cells under stress.

We demonstrated the protective role of AoDHN1 in *E. coli* cells expressing the protein and subjected to salinity and drought stress conditions. The significant increase in cell density at 6 h without any treatment compared to control cells without AoDHN1 (Figure 8A) suggests the overall growth advantage provided by the protein. Thus, our data suggest that AoDHN1 may help in stabilizing the cells during abiotic stress conditions even in *A. officinalis.*

## Conclusions

In conclusion, we have identified several novel genes that are specifically expressed in the salt gland-rich tissues of *A. officinalis*. A gene interactive network has been generated based on the ESTs identified from SH analysis. A dehydrin gene *AoDHN1* that is highly expressed in salt gland cells was identified and cloned. *AoDHN1* was up-regulated in response to drought and salt treatments and was shown to play an important role in alleviating salt and drought stresses. *A. officinalis*, which is an obligate halophyte, may use this dehydrin protein to protect the cellular components such as enzymes and other macromolecules from dehydration damage caused by physiological drought. The results from our study have helped to identify a key protective protein that represents one of the numerous players in the complex molecular mechanism underlying salt tolerance in mangroves.

## Methods

### Plant materials and growth conditions

*Avicennia officinalis* stem cuttings from field grown trees and propagules were collected from mangrove swamps near the Berlayer Creek, Singapore (1.27°N; 103.80°E) and Sungei Buloh Wetland Reserve, Singapore (1.43°N; 103.717°E). Propagules were grown in potting mix (Far East Flora, Singapore) in green house condition (25–35°C, 60–90% relative humidity; 12 h photoperiod) watered every alternate day with NaCl-free water.

### Tissue preparation and subtractive hybridization

*A. officinalis* leaves collected from several field-grown trees were used for SH. The upper epidermal peels were separated from the mesophyll as described earlier [25] and kept frozen separately in -80°C until RNA was extracted. Total RNA was isolated using Qiagen plant RNAeasy kit, from the upper epidermal peels and mesophyll cells. SH was performed using the upper epidermal peel RNA (rich in salt glands) as the tester and mesophyll tissue RNA as the driver. SH service was obtained from First BASE Laboratories (www.base-asia.com).

### Data cleaning and gene annotation

The ESTs of the raw mangrove data were cleaned up to remove the duplicate sequences using the program LAST (http://last.cbrc.jp/). The aligned sequences having 99% identity were created using the parameter ‘lastal -r1 -q99 -a0 -b99 -e150’. The duplicate sequences were then removed based on 100% identity.

Putative functions were assigned for the unique genes by blasting the unique ESTs against all genes including experimentally verified, predicted and hypothetical genes of the following plant species : *Arabidopsis thaliana* and *Oryza sativa, Glycine max*, and *Populus trichocarpa* by NCBI- BLASTX Algorithim [66]. For this purpose, the cDNA sequences of these plant species were downloaded from Plant GDB database (http://www.plantgdb.org/). Since the cDNA sequences were not available for *Avicennia marina*, the EST sequences were used to find out the gene similarity. Blast results having more than 70% identity with an *e*-value cut off of 1e^−10^ and blast scores more than 50 bits were considered as significant.

### Cloning *AoDHN1* cDNA and genomic DNA fragment

Full length CDS of *AoDHN1* was obtained using SMART RACE cDNA Amplification Kit (Clontech). Sequences were amplified using the primers 5^′^- GTCTTCGGAGGACGATGG 3^′^ (forward) for 3′ RACE and 5^′^-CCATCGTCCTCCGAAGAC-3^′^ (reverse) for 5′RACE. Corresponding genomic DNA sequence was obtained by amplifying the genomic DNA with 5^′^-ATGTCAGAGTACGGCGA-3^′^ (forward) and 5^′^-ATGGTGGCCTCCGGGCA-3^′^ (reverse) primers. The fragments generated by RACE and genomic DNA amplification were cloned into pGEMT-Easy vector (Promega) and sequenced.

### Sequence comparison and structure prediction of DHNs

Nucleotide sequences were translated to protein using ExPASy translate (http://web.expasy.org/translate/) The sequence alignment was done using ClustalW2 multiple alignment (http://www.ebi.ac.uk/Tools/msa/clustalw2/) and representation was done using BoxShade Server (http://www.ch.embnet.org/software/BOX_form.html) which highlight identities and similarities in protein sequence. Secondary structure of the proteins was predicted using PSIPRED (http://bioinf.cs.ucl.ac.uk/psipred/). Three dimensional structure of the protein was predicted using iTASSER (http://zhanglab.ccmb.med.umich.edu/I-TASSER/).

### Treatment protocols

For tissue-specific expression, leaf, stem and root tissues from two-month-old *A. officinalis* plants were used. Plants that were transferred to pots containing sand and allowed to adapt for two days by watering with half-strength Hoagland’s solution were used for this purpose. These plants were later subjected to 500 mM NaCl treatment (also in half-strength Hoagland’s solution) to study the expression kinetics of *AoDHN1* in roots and leaves.

To study the expression pattern of *AoDHN1 *during abiotic stress, leaf-discs from two-month-old *A. officinalis* plants were used. Fully expanded leaves were chosen to make approximately 6 mm diameter leaf-discs. For drought treatment, the leaf-discs were placed on silica beads in a petri dish and dried in a laminar air flow. For ABA treatment, the leaf-discs were incubated in 2 μM ABA while for salt treatment leaf-discs were incubated in 200 mM NaCl. Water-treated leaf-discs were used as the control. Leaf-discs were vacuum infiltrated for 5 min in their respective treatment solutions before they were incubated to various time periods. The duration of treatments was 30 min, one and two hours for the leaf-disc experiment.

### qRT-PCR analysis

Expression analysis was performed by qRT-PCR for several genes including *AoDHN1*. Reactions were performed on cDNA, prepared from various *A. officinalis* tissues. Total RNA was isolated using ‘RNeasy Plant Kit’ (QIAGEN). cDNA was prepared using ‘MAXIMA First Strand cDNA Synthesis Kit’ (Fermentas). Reaction was performed with ‘KAPA SYBR FAST qPCR Kit’ (KAPA Biosystems) using the ‘StepOne™ Real-Time PCR Systems’ (Applied Biosystems). All qRT-PCR data were generated from three independent biological replicates, each with three technical replicates (n = 3). Relative quantification of expression was determined using ‘StepOne Software’ (v2.1). Primers were designed using NCBI web-tool (http://www.ncbi.nlm.nih.gov/tools/primer-blast/) and the list of SH qRT-PCR primers have been provided in Additional file 3. Constitutively expressed *Ubiquitin 10* was used as internal control and the primer sequences were 5^′^-CGCCGGCAAGCAGCTAGAGG -3^′^(forward) and 5^′^-ACCACGGAGCCTGAGGACCA-3^′^ (reverse) for *Ubiquitin 10* (AT4G05320) (250 bp). The primer sequences used to amplify *AoDHN1* are 5′-GACACCACTGGAGCGT-3′ (forward) 5′-TCCGTAGTTCCGTACC-3′ (reverse).

### *In situ* hybridization

*In situ* hybridization was carried out using leaf sections obtained from two-month-old green-house grown-plants following a published protocol [67] with minor modifications. For sense and anti-sense DIG-labeled RNA probe synthesis, the pGEM-T Easy vector (Promega) containing RT-PCR-amplified inserts using (5^′^-ATGTCAGAGTACGGCGA-3^′^ (forward) and 5^′^-ATGGTGGCCTCCGGGCA-3^′^ (reverse) primers were linearized and *in vitro* transcribed using DIG-RNA Labeling Kit (Roche). Representative photographs are shown from at least five independent replicates examined.

### Sub-cellular localization of *AoDHN1* in Arabidopsis mesophyll protoplasts

The coding region of *AoDHN1* was amplified using 5^′^-CTCGAGATGTCAGAGTACGGCGA-3^′^ (forward) and 5^′^-CCCGGGATGGTGGCCTCCGGGCA-3^′^ (reverse) primers with XhoI and SmaI restriction sites cloned into pGreen (HY105 backbone) containing mGFP (cloned with SpeI and XbaI), to generate *pGreen-35S::AoDHN1-GFP*. Also, *pPLV06 UBI::NLS*_*SV40*_*-YFP* was used as the nuclear marker [68].

Leaf mesophyll protoplasts were isolated from 3- to 4-week-old wild-type *Arabidopsis thaliana* (Col-0) plants [69] and transfected using 20 μg of plasmid DNA each (*pGreen-35S::AoDHN1-GFP* and *pPLV06 UBI::NLS*_*SV40*_*-YFP*). Images were acquired 12 h to16h after transfection using a Carl Zeiss Axiovert 200 M confocal laser scanning microscope ("http://www.zeiss/de/axiovert200.) with excitation at 488 nm (for GFP) and 514 nm (for YFP). Images of the signals obtained from protoplasts were determined using Zen 2012 SP2 software from Carl Ziess (Representative photographs are shown from at least eight independent protoplasts).

### Southern blotting

Genomic DNA was extracted from *Avicennia officinalis* leaves by CTAB method [70] and was digested with restriction enzymes (EcoRI, HindIII and KpnI). Full length probe was amplified using the 5^′^-ATGTCAGAGTACGGCGA-3^′^ (forward) and 5^′^-ATGGTGGCCTCCGGGCA-3^′^ (reverse) primers. *AoDHN1* probes were synthesized using Roche PCR DIG labelling kit. Southern blot was carried out following manufacturer’s protocol (http://www.roche-applied-science.com).

### Functional assay of AoDHN1 in *E. coli*

The coding region of *AoDHN1* was amplified using 5^′^-GATCCATGTCAGAGTACGGCGA-3^′^ (forward) and 5^′^-CTCGAGTTAATGGTGGCCTCCGGG-3^′^ (reverse) primers with BamHI and XhoI restriction sites. It was cloned into the over-expression vector *pGEX-6P-1* containing GST tag, to generate the *AoDHN1-GST* fusion construct. This fusion plasmid was introduced into *E. coli* BL21 cells by heat shock method. The transformed BL21 cells were first grown to log phase as determined by OD_600_ = 0.5. Equal volume of these cultures were transferred to sterile culture tubes with 10 ml of LB medium containing 100 μg/ml ampicillin, 1 mM IPTG (final concentration) with varying NaCl and PEG concentrations (Additional file 4 and Additional file 5). Finally, growth assay with 400 mM NaCl, 10% PEG (PEG 4000) and 500 mM mannitol treatments (final concentration) were carried out. The cells were allowed to grow at 37°C and the growth was monitored at OD_600_ at specific time points.

## Additional files

Additional file 1
**Expression analysis of ESTs with less than twofold difference in expression or with higher expression in the mesophyll tissue.** **(A)** qRT-PCR analysis of ESTs that showed less than twofold difference in expression in salt gland-rich tissue compared to mesophyll tissue. *NAC domain containing protein 32 (NAC), Ubiquitin conjugating enzyme 2 (UC2), Serine/Threonine-protein kinase (ST), Casein kinase (CK2), Xylem Cysteine Peptidase 2 (XCP2), Transcription factor HBP1b (HBP), Trypsin family protein (T), Phospholipase D (PD), Serine/Arginine-rich protein spicing factor 34b (SA), Syringolide-induced protein 19-1-5 (SIP), Trehalose 6-phosphate synthase S6 (TPS).* **(B)** qRT-PCR analysis of ESTs that showed less expression in salt gland-rich tissue than in mesophyll tissue. *Mitochondrial Rho GTPase (MR), Plasma membrane H* ^*+*^ *ATPase (HATPase), Salt-inducible Zinc Finger 2 (ZF2), Vacular ATP synthase subunit D (VATD), Auxin signalling F-box 2 (AF2), Peroxidase (PS), Glutamate synthase (GS), Protein translation factor SUI1 homolog (PTF), 26S protein regulatory subunit 4 homolog (PRS), Disease resistance (DR), AP2 domain containing transcription factor (AP2), Cytochrome –C Oxidase (CCO), Arginine decarboxylase (AD).* Data are mean ± SE (n = 3). RQ – Relative quantification. Additional file 2
**Secondary structure of AoDHN1, AoDHN2 and AmDHN1.** Secondary structure of the dehydrins was predicted using PSIPRED (http://bioinf.cs.ucl.ac.uk/psipred/). All the three dehydrins **(A)** AoDHN1 **(B)** AmDHN1 **(C)** AoDHN2 predominantly showed intrinsically unstructured portion in the protein except two α-helices at the two K segments towards the C-terminus. Additional file 3
**List of primers used for qRT-PCR analysis and cloning of** ***AoDHN1.*** qRT-PCR primers were designed using NCBI primer design web-tool (http://www.ncbi.nlm.nih.gov/tools/primer-blast/) for 34 ESTs that were identified from SH. Additional file 4
**Effect of different salt concentrations on growth of** ***E. coli*** **cells expressing AoDHN1.** Differences in cell densities of *E. coli* cells expressing AoDHN1 and control *E. coli* cells when subjected to varying NaCl concentrations over a period of time **(A)** 200 mM NaCl treatment **(B)** 300 mM NaCl treatment **(C)** 400 mM NaCl treatment and **(D)** 500 mM NaCl treatment. *E. coli* cells expressing AoDHN1 showed a significant increase in cell density compared to control cells without AoDHN1. Treatment with 400 mM NaCl showed significant difference in the cell densities of *E. coli* cells and this concentration of NaCl was chosen for detailed analysis. Data are mean ± SE (n = 3). Asterisks indicate a significant difference in cell densities as indicated by Student’s *t*-test (*p* < 0.05). OD – Optical Density. Additional file 5
**Effect of different concentrations of PEG on growth of** ***E. coli*** **cells expressing AoDHN1.** Differences in cell densities of *E. coli* cells expressing AoDHN1 and control *E. coli* cells when subjected to varying PEG concentrations over a period of time **(A)** 5% PEG treatment **(B)** 10% PEG treatment **(C)** 15% PEG treatment and **(D)** 20% PEG treatment. *E. coli* cells expressing AoDHN1 showed a significant increase in cell density compared to control cells without AoDHN1. Treatment with 10% PEG showed significant difference in the cell densities of *E. coli* cells and this concentration of PEG treatment was chosen for detailed analysis. Data are mean ± SE (n = 3). Asterisks indicate a significant difference in cell densities as indicated by Student’s *t*-test (*p* < 0.05). OD – Optical Density.

## Abbreviations

SH: Subtractive hybridization
ESTs: Expressed sequence tags
qRT-PCR: Quantitative real-time PCR
*AoDHN1*: *Avicennia officinalis Dehydrin1*
RACE: Rapid amplification of cDNA ends
REVIGO: REduce VIsualize gene ontology
ABA: Abscisic acid
GO: Gene ontology
NLS: Nuclear localization sequence
LEA protein: Late embryogenesis abundant protein
IPTG: Isopropyl β-D-1-thiogalactopyranoside
PEG: Poly ethylene glycol
GFP: Green fluorescence protein
YPF: Yellow fluorescence protein
