# Supplementary figures and images for: Identification of salt gland-associated genes and characterization of a dehydrin from the salt secretor mangrove Avicennia officinalis
Source: BMC Plant Biol. 2014 Nov 18;14:291. doi: 10.1186/s12870-014-0291-6 (PMC4247641; doi:10.1186/s12870-014-0291-6)

**A**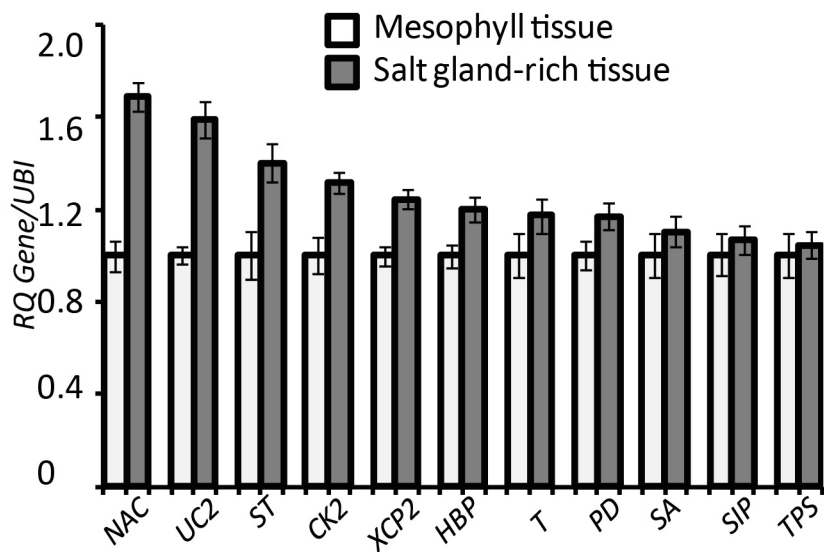**B**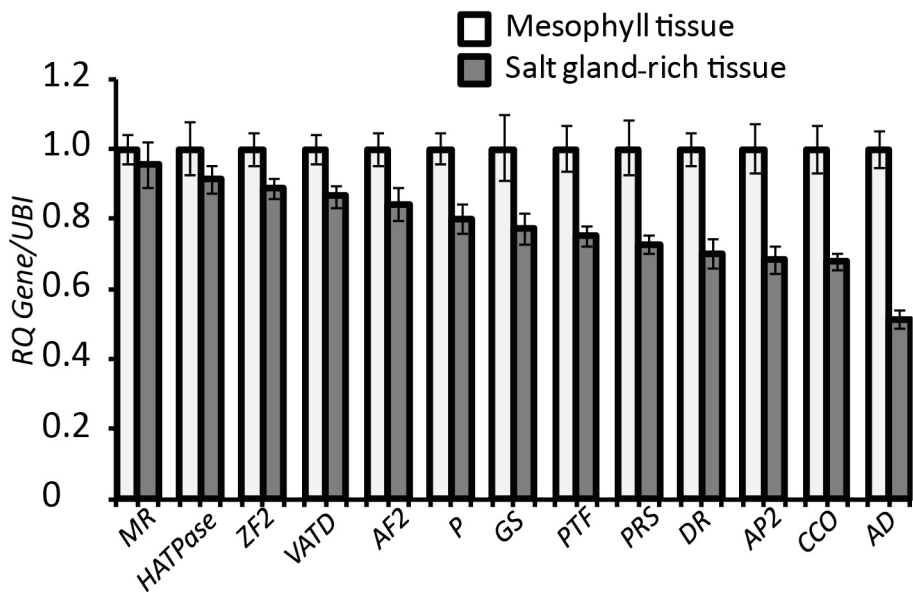

Supplement: Additional file 1 — Expression analysis of ESTs with less than twofold difference in expression or with higher expression in the mesophyll tissue. (A) qRT-PCR analysis of ESTs that showed less than twofold difference in expression in salt gland-rich tissue compared to mesophyll tissue. NAC domain containing protein 32 (NAC), Ubiquitin conjugating enzyme 2 (UC2), Serine/Threonine-protein kinase (ST), Casein kinase (CK2), Xylem Cysteine Peptidase 2 (XCP2), Transcription factor HBP1b (HBP), Trypsin family protein (T), Phospholipase D (PD), Serine/Arginine-rich protein spicing factor 34b (SA), Syringolide-induced protein 19-1-5 (SIP), Trehalose 6-phosphate synthase S6 (TPS). (B) qRT-PCR analysis of ESTs that showed less expression in salt gland-rich tissue than in mesophyll tissue. Mitochondrial Rho GTPase (MR), Plasma membrane H + ATPase (HATPase), Salt-inducible Zinc Finger 2 (ZF2), Vacular ATP synthase subunit D (VATD), Auxin signalling F-box 2 (AF2), Peroxidase (PS), Glutamate synthase (GS), Protein translation factor SUI1 homolog (PTF), 26S protein regulatory subunit 4 homolog (PRS), Disease resistance (DR), AP2 domain containing transcription factor (AP2), Cytochrome –C Oxidase (CCO), Arginine decarboxylase (AD). Data are mean ± SE (n = 3). RQ – Relative quantification. [file 12870_2014_291_MOESM1_ESM.pdf]

A)

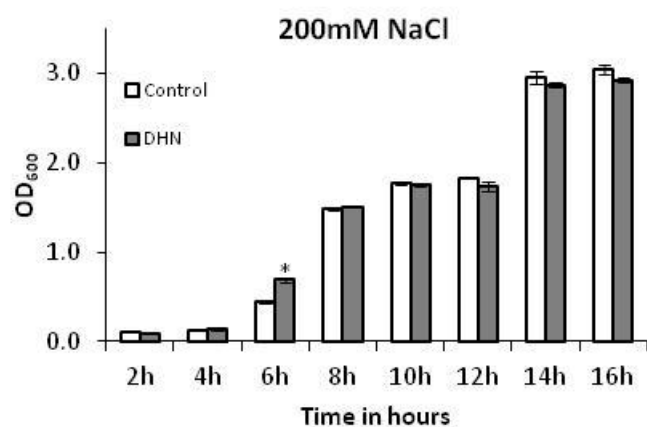

B)

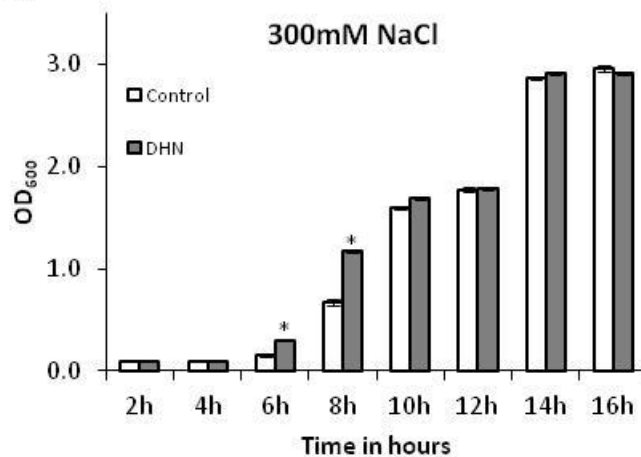

C)

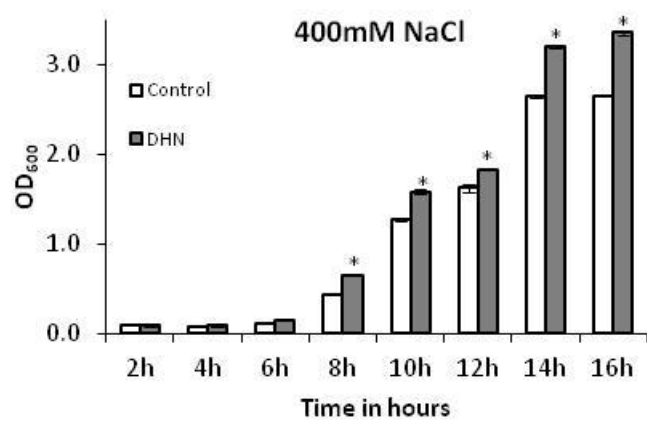

D)

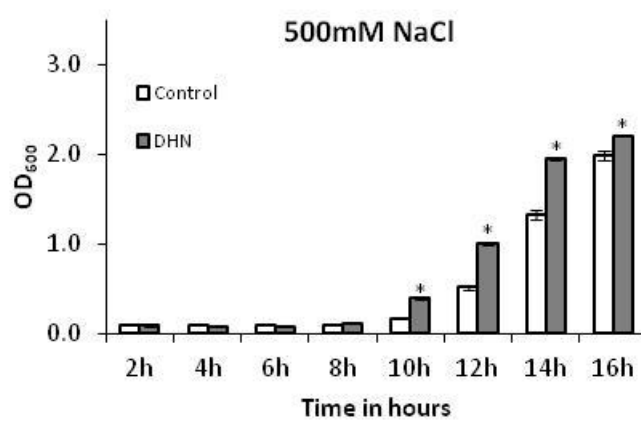

Supplement: Additional file 4 — Effect of different salt concentrations on growth of E. coli cells expressing AoDHN1. Differences in cell densities of E. coli cells expressing AoDHN1 and control E. coli cells when subjected to varying NaCl concentrations over a period of time (A) 200 mM NaCl treatment (B) 300 mM NaCl treatment (C) 400 mM NaCl treatment and (D) 500 mM NaCl treatment. E. coli cells expressing AoDHN1 showed a significant increase in cell density compared to control cells without AoDHN1. Treatment with 400 mM NaCl showed significant difference in the cell densities of E. coli cells and this concentration of NaCl was chosen for detailed analysis. Data are mean ± SE (n = 3). Asterisks indicate a significant difference in cell densities as indicated by Student’s t-test (p < 0.05). OD – Optical Density. [file 12870_2014_291_MOESM4_ESM.pdf]

A)

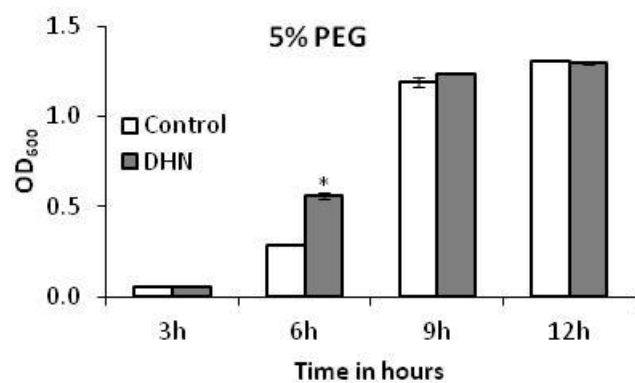

B)

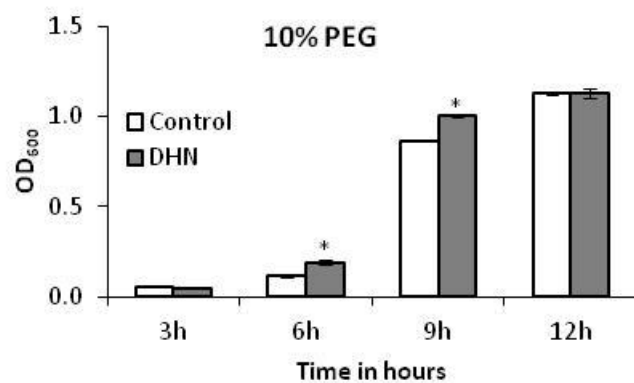

C)

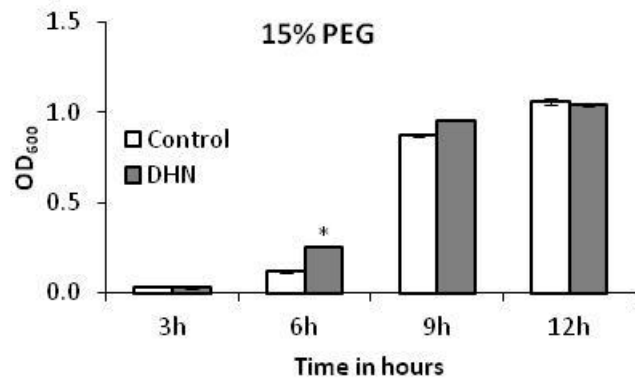

D)

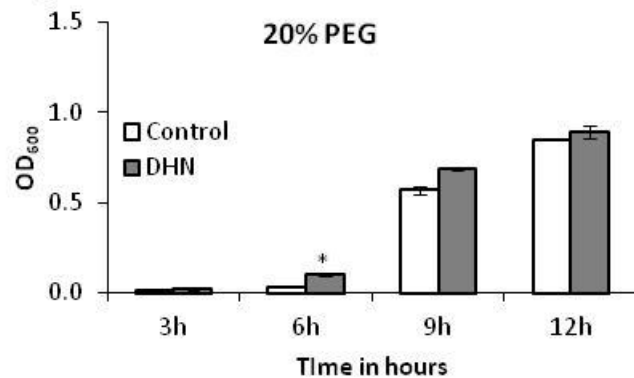

Supplement: Additional file 5 — Effect of different concentrations of PEG on growth of E. coli cells expressing AoDHN1. Differences in cell densities of E. coli cells expressing AoDHN1 and control E. coli cells when subjected to varying PEG concentrations over a period of time (A) 5% PEG treatment (B) 10% PEG treatment (C) 15% PEG treatment and (D) 20% PEG treatment. E. coli cells expressing AoDHN1 showed a significant increase in cell density compared to control cells without AoDHN1. Treatment with 10% PEG showed significant difference in the cell densities of E. coli cells and this concentration of PEG treatment was chosen for detailed analysis. Data are mean ± SE (n = 3). Asterisks indicate a significant difference in cell densities as indicated by Student’s t-test (p < 0.05). OD – Optical Density. [file 12870_2014_291_MOESM5_ESM.pdf]
