# Supplementary material for: Identification of salt gland-associated genes and characterization of a dehydrin from the salt secretor mangrove Avicennia officinalis
Source: BMC Plant Biol. 2014 Nov 18;14:291. doi: 10.1186/s12870-014-0291-6 (PMC4247641; doi:10.1186/s12870-014-0291-6)
Supplement: Additional file 2 — Secondary structure of AoDHN1, AoDHN2 and AmDHN1. Secondary structure of the dehydrins was predicted using PSIPRED (http://bioinf.cs.ucl.ac.uk/psipred/). All the three dehydrins (A) AoDHN1 (B) AmDHN1 (C) AoDHN2 predominantly showed intrinsically unstructured portion in the protein except two α-helices at the two K segments towards the C-terminus. [file 12870_2014_291_MOESM2_ESM.pdf]

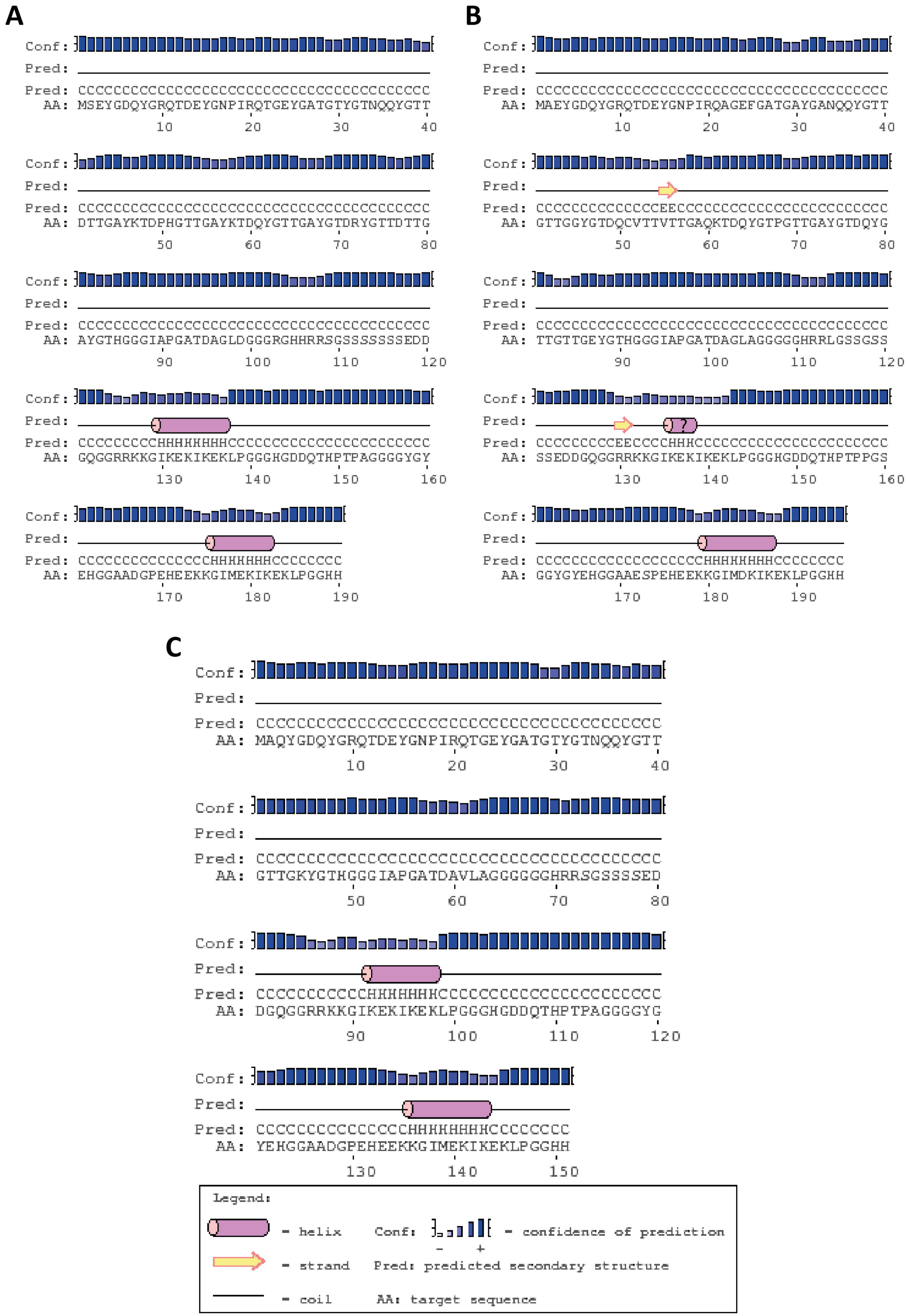
A
Pred: CCCCCCCCCCCCCCCCCCCCCCCCCCCCCCCCCCCCCCCC
AA: MSEYGDQYGRQTDEYGNPIRQTGEYGATGTYGTNQQYGTT
Pred: CCCCCCCCCCCCCCCCCCCCCCCCCCCCCCCCCCCCCCCC
AA: DTTGAYKTDPHGTTGAYKTDQYGTTGAYGTDRYGTTDTTG
Pred: CCCCCCCCCCCCCCCCCCCCCCCCCCCCCCCCCCCCCCCC
AA: AYGTHGGGIAPGATDAGLDGGGRGHHRRSGSSSSSSSEDD
Pred: CCCCCCCCCHHHHHHHHHCCCCCCCCCCCCCCCCCCCCCC
AA: GQGGRRKKGIKEKIKEKLPGGGHGDDQTHPTPAGGGGYGY
Pred: CCCCCCCCCCCCCCCHHHHHHHHCCCCCCC
AA: EHGGAADGPEHEEKKGIMEKIKEKLPGGHH
B
Pred: CCCCCCCCCCCCCCCCCCCCCCCCCCCCCCCCCCCCCCCC
AA: MAEYGDQYGRQTDEYGNPIRQAGEFGATGAYGANQQYGTT
Pred: CCCCCCCCCCCCCCEECCCCCCCCCCCCCCCCCCCCCCCC
AA: GTTGGYGTDQCVTTVTTGAQKTDQYGTPGTTGAYGTDQYG
Pred: CCCCCCCCCCCCCCCCCCCCCCCCCCCCCCCCCCCCCCCC
AA: TTGTTGEYGTHGGGIAPGATDAGLAGGGGGHRRLGSSGSS
Pred: CCCCCCCCCEECCCCHHHCCCCCCCCCCCCCCCCCCCCCC
AA: SSEDDGQGGRRKKGIKEKIKEKLPGGGHGDDQTHPTPPGS
Pred: CCCCCCCCCCCCCCCCCCCHHHHHHHHCCCCCCCC
AA: GGYGYEHGGAAESPEHEEKKGIMDKIKEKLPGGHH
C
Pred: CCCCCCCCCCCCCCCCCCCCCCCCCCCCCCCCCCCCCCCC
AA: MAQYGDQYGRQTDEYGNPIRQTGEYGATGTYGTNQQYGTT
Pred: CCCCCCCCCCCCCCCCCCCCCCCCCCCCCCCCCCCCCCC
AA: GTTGKYGTHGGGIAPGATDAVLAGGGGGHRRSGSSSSED
Pred: CCCCCCCCCCCHHHHHHHCCCCCCCCCCCCCCCCCCCCCC
AA: DGQGGRRKKGIKEKIKEKLPGGGHGDDQTHPTPAGGGGYG
Pred: CCCCCCCCCCCCCCCHHHHHHHHHCCCCCCC
AA: YEHGGAADGPEHEEKKGIMEKIKEKLPGGHH
Legend:
- helix
Conf: - confidence of prediction
- strand
Pred: predicted secondary structure
- coil
AA: target sequence
