# Supplementary material for: Identification of salt gland-associated genes and characterization of a dehydrin from the salt secretor mangrove Avicennia officinalis
Source: BMC Plant Biol. 2014 Nov 18;14:291. doi: 10.1186/s12870-014-0291-6 (PMC4247641; doi:10.1186/s12870-014-0291-6)
Supplement: Additional file 3 — List of primers used for qRT-PCR analysis and cloning of AoDHN1. qRT-PCR primers were designed using NCBI primer design web-tool (http://www.ncbi.nlm.nih.gov/tools/primer-blast/) for 34 ESTs that were identified from SH. [file 12870_2014_291_MOESM3_ESM.pdf]

Sl. No.	Gene Abbreviation	Forward primer sequence 5'-3'	Reverse primer sequence 5'-3'
1	***MR***	GAGGCCGAATTGAGACCACA	GCAGCCGTTTTGTTGGGAAT
2	***LR***	GGGACCGTCGAAGAGTTTGT	GCTGCCCCATCATAGCTGTT
3	***ST***	TCTGCAATGCCCTGTCTTGA	TGGCATCTATACTGGCAGCAA
4	***SA***	CGTTCAAGGTCTCGCTCAGT	CTTTTGCTTGCACTGCGTCT
5	***T***	GGTAGTAGCCTGCAACCCAG	CCATTAGGTGGCCAGCTCTC
6	***AD***	TCAACGTCAGCGAGAAGCTC	GCAGGCAATCCAGCATTTCA
7	***ACL***	AGGAACCATGGCTCTCATCAA	ACAAATGCAGCAACTCCAGC
8	***PD***	GCCTAATTTCACTGGGGCTG	TTCCCACCTTGCTTTCTCCA
9	***SIP***	CAGCAACGGATGGCTGAATG	CTTGGCATCGGTGCAGTAGT
10	***TPS***	CTGTGCTGGTGCAGATCGTA	GGCTTCCCAAGCTCCTCATT
11	***ACC***	GCTTGAAGCTGTTCAGTCCG	AGTTTCTCCAACTGGCCTGC
12	***PS***	TGCTGGAGCCGATTACTGTG	GTGTCTAGCTGCCAAGACCT
13	***AF2***	TGCGGTGACCAATCTACCAC	CCTTCGTCACTATCCCCAGC
14	***HBP***	GGGGGCCGTTGATCTTTTTC	TCAACAGGAACTCAGCACGA
15	***AP2***	CCCCCTGTCCAACAAGAGTC	GGCGAGGAGTTCAAAGGCTA
16	***RP***	CTCAGTGGCACATGGAACCT	TGGATCAATCCGCCTCAACG
17	***DR***	TGTCTGCCCGAAAGATGGTT	ATCCTTTCAAGGGAGTGGAGA
18	***CK2***	ACTGGGGACTTGCTGAGTTT	TCCAGCAAACATACAGCCCA
19	***XCP2***	GAAATCCGGCCTCTGTGGAA	AGACATTAGGCGGTGCAACA
20	***GS***	GCCACGACCTCCTTTGAAGA	AGCACGCTCAAATACCGTCA
21	***KCS***	CAGAAGCCAAAGGCAGGGTA	CTTGCACTCTTGAGCTGGGA
22	***PTF***	GCTGCAATGGAACTGTGGTT	TCCTGCCTGAACGAGAAACG
23	***UC2***	ACCCTGCACCAACAAGACTC	GGGTCTTGCTGCAACCTCTT
24	***AQP***	GCTTTCCCTGACCAGGGCTC	GCCCCTCCTTTAAGCCTCTG
25	***PRS***	CGCCGATGAGTGTTGGAAAC	AAGAATCGCACATCCAGGCT
26	***CCO***	GAGTCGAGGGTGTAATGCCG	TCCAAAGGGACCAGATGGGT
27	***TR***	ATTCTAAGCGCCGAGACTGG	AGAGGAGGACTAACAGCCCA
28	***DHN***	GACACCACTGGAGCGT	TCCGTAGTTCCGTACC
29	***NAC***	TCGACGCATCTGACTCGTTT	GCTCACTCCACTTGGGTTCA
30	***R2R3***	TCTCAGATACGGCCACTGGA	TTCCCTCGTTTCACACCTGG
31	***ZF2***	GATTTTCGCAGGGGTGCTTG	TGCCATCTTTGCACAGCCTA
32	***ABC***	GGTCACTCTCAGCAACGGAA	GTCGTCCAGAAAGGGCATCA
33	***VATD***	TGCGGCACTTAAGGTTCGAT	GCAAGCTTGAACCTGCTGAC
34	***HATPase***	CAAAGAACTCTCCACGGGCT	CTTGCCATTTCAGCTCGACG
